# Thermal Conductivity and Dielectric Properties of EP Composites Enhanced by BNNS-AgNP Synergistic Doping

**DOI:** 10.3390/nano16120704

**Published:** 2026-06-08

**Authors:** Haibin Zhou, Jun Deng, Zhicheng Xie, Zhicheng Pan, Yanjie Cui, Dong Yue, Yu Feng, Mingze Zhang, Minghe Chi, Xunjun He

**Affiliations:** 1 Electric Power Research Institute of Extra High Voltage Transmission Company, China Southern Power Grid Co., Ltd., Guangzhou 510530, China15129363787@139.com (Y.C.); 2 School of Electrical and Electronic Engineering, Harbin University of Science and Technology, Harbin 150080, China

**Keywords:** EP, BNNSs-AgNPs hybrid fillers, synergistic interaction, thermal conductivity, dielectric constant

## Abstract

To meet the growing demand for materials combing high thermal conductivity and electrical insulation, we developed epoxy (EP) composites filled with zero-dimensional (0D) silver nanoparticles (AgNPs) and two-dimensional (2D) boron nitride nanosheets (BNNSs). This hybrid filler system synergistically enhances both thermal conductivity and dielectric properties, while retaining excellent electrical insulation. With only 1 wt% AgNPs and 15 wt% BNNSs, the composite achieved a dielectric constant of 4.17 at 100 Hz, outperforming pure EP. At 30 wt% BNNSs and the same AgNP loading, the in-plane and out-of-plane thermal conductivities reached 3.02 and 0.41 W·m^−1^·K^−1^, respectively, along with improved thermal stability. Moreover, the composite exhibited an electrical conductivity below 10^−9^ S/cm at 1000 Hz, confirming that the minimal metal filler content negligibly affects insulation. Thus, this work offers a feasible strategy for designing next-generation high-performance composites using 0D/2D hybrid fillers, highlighting their promising potential for advanced electronic packaging.

## 1. Introduction

Epoxy resins (EPs) are widely used in electronic packaging due to their excellent electrical insulation and processability [1,2,3,4]. However, their intrinsic low thermal conductivity (~0.2 W·m^−1^·K^−1^) limits heat dissipation, necessitating performance enhancement [5]. To improve the thermal conductivity of EP composites, a common strategy involves incorporating highly thermally conductive fillers and/or constructing 3D pathways [6,7,8,9]. Among these, 2D boron nitride nanosheets (BNNSs) are particularly attractive because of their high in-plane thermal conductivity (~300 W·m^−1^·K^−1^) and outstanding electrical insulation [10,11]. For instance, Tang et al. reported that the thermal conductivity of BNNS/EP composites increases with the filler loading, whereas the dielectric behavior exhibits more complex trends [12]. Long et al. observed that BNNS addition improves the thermal conductivity of the composites but reduces the dielectric constant [13]. Wang et al. constructed a 3D BNNS thermal conduction network, achieving over a 50-fold improvement in the thermal conductivity at 53.92 wt% filler loading relative to neat epoxy [14]. Nevertheless, BNNSs tend to aggregate, leading to significant interfacial thermal resistance and deteriorated thermal and mechanical properties [15].

To overcome these limitations in single-filler systems, hybrid-filler strategies have been widely explored. Combining BNNSs with other components can improve dispersion, strengthen interfacial interactions, and facilitate efficient 3D thermal networks [16,17,18,19,20]. For example, Fu et al. prepared BNNS/PA6 composites using a template-free freeze-drying method, achieving significantly better thermal conductivity and dielectric properties than those of neat epoxy or randomly dispersed BNNS/EP composites [21]. Wang et al. reported epoxy composites co-filled with BNNS/Al_2_O_3_, exhibiting increased out-of-plane thermal conductivity and dielectric performance. Zhao et al. utilized BN microspheres (BNMSs) and SiO_2_-coated SiC nanoparticles as hybrid fillers to achieve both a high thermal conductivity and high dielectric constant in epoxy composites [22]. Zhang et al. fabricated epoxy composites with AgNW/GO hybrid networks using solution mixing, improving thermal conductivity and impact strength [23]. Despite these advances, many such methods require multi-step surface functionalization, increasing fabrication complexity and cost [24,25,26]. Therefore, developing a relatively simple hybrid design capable of effectively enhancing thermal transport while preserving dielectric reliability remains a compelling research objective.

Recently, 0D silver nanoparticles (AgNPs) have attracted considerable interest due to their exceptionally high intrinsic thermal conductivity [27,28,29]. AgNPs can serve as nanoscale “thermal bridges” and interfacial modifiers, and have been widely employed to enhance heat transport in polymer composites [30,31,32]. However, existing studies typically pre-form BNNS/Ag hybrids through liquid-phase exfoliation followed by in situ reduction of Ag^+^ on BNNS surfaces, involving multiple steps and potential impurities, or rely on functionalizing BNNSs to anchor AgNPs and improve dispersion. Moreover, Ag is primarily used as “thermal bridges” at BNNS junctions to maximize thermal conductivity. In this paper, we investigate the synergistic effects of directly co-doping BNNSs and AgNPs into EP composites using a relatively simple fabrication process. Our results show that a small amount of AgNPs enhances both the thermal conductivity and dielectric properties of BNNS/EP composites while maintaining adequate electrical insulation performance. Unlike previous studies [33,34], our strategy adopts direct filler blending without any functionalization, and Ag mainly serves as a local interface modifier to balance thermal conductivity and electrical insulation. Therefore, this work provides practical insights and a foundation for developing next-generation epoxy-based packaging materials that combine high thermal conductivity with reliable dielectric properties.

## 2. Preparation and Characterization of AgNp-BNNS/EP Composites

### 2.1. Preparation Process

**Figure 1:**
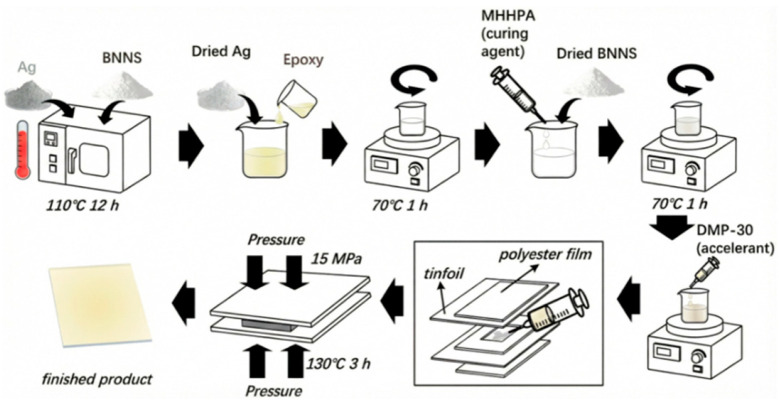
Schematic diagram of the preparation process of AgNP-BNNS/EP composites.

To fabricate the AgNp-BNNS/EP composites, all materials, including hexagonal boron nitride (h-BN from Shanghai Yaotian Chemical Co., Ltd., Shanghai, China), spherical silver powder (AgNPs from Suzhou Lengshi New Materials Co., Ltd., Suzhou, China), epoxy resin E-51 (EP from Nantong Xingchen Synthetic Materials Co., Ltd., Nantong, China), methylhexahydrophthalic anhydride (MHHPA from Puyang Huicheng Electronic Material Co., Ltd., Puyang, China), and 2,4,6-tris (dimethylaminomethyl) phenol (DMP-30 from Nantong Xingchen Synthetic Materials Co., Ltd., Nantong, China), were used as received without further pre-treatment. Figure 1 schematically illustrates the preparation process. First, exfoliated BNNSs and AgNPs (0D spherical or 2D flakes) were vacuum-dried at 110 °C for 12 h. A specified amount of AgNPs (0.5 or 1 wt% relative to EP) was then added to 3 g of uncured EP and magnetically stirred at 70 °C for 1 h, where the elevated temperature improved EP fluidity to promote uniform dispersion of AgNPs. Subsequently, MHHPA and BNNSs (0 wt%, 10 wt%, 15 wt%, 20 wt%, 25 wt%, or 30 wt% relative to EP) were added, and stirring continued at 70 °C for another hour to ensure complete dispersion of the BNNSs. Next, 2–3 drops of DMP-30 were introduced under continuous stirring and mixed for an additional 5 min. The well-mixed liquid was then quickly drawn into a syringe and injected into the cavity of the middle layer of a three-layer polyester film mold (top, middle, bottom, and middle layer, with a hollowed cavity), indicated by a black border in Figure 1. Aluminum foil inserted between the polyester films prevented adhesion of the cured epoxy to the film surfaces. Finally, the mold was hot-pressed in a flat vulcanizing press at 130 °C under 15 MPa for 3 h, and the composite was obtained after cooling to room temperature. Moreover, this hybrid filler strategy offers several distinct advantages over existing studies [35,36]. First, fabrication is simple: all composites are prepared by mechanical stirring at 70 °C followed by hot-pressing at 130 °C, without any pre-treatment, high-shear mixing, or solution processing, ensuring scalability. Second, no surface functionalization of h-BN or AgNPs is applied, reducing cost and complexity while achieving adequate dispersion. Third, the hybrid system enables simultaneous dielectric and thermal optimization: insulating BNNS layers act as physical barriers preventing AgNP contact and percolation, preserving excellent electrical insulation, while AgNPs enhances thermal conduction, an uncommon synergy in single-filler systems. Fourth, the composites retain high breakdown strength close to that of neat epoxy even at 30 wt% BNNSs, as BNNS barriers suppress charge transport and electrical tree propagation, with no conductivity surge or premature failure.

### 2.2. Characterization Methods

The morphology and elemental distribution of the AgNP-BNNS/EP composites were examined using a field-emission scanning electron microscope (SEM, Zeiss Supra 55, Germany) equipped with an energy-dispersive X-ray spectroscopy (EDS) detector. SEM imaging was performed at an accelerating voltage of 10 kV and a working distance of 8 mm. To avoid charging effects, cross-sectional samples were sputter-coated with a thin (~5 nm) gold layer. EDS mapping was conducted at the same voltage with an acquisition time of 15 min to ensure adequate signal-to-noise ratios for Ag, B, and N. Thermal conductivity was measured using a TPS 2500 S thermal constant analyzer (Hot Disk AB, Sweden), which enables high-precision determination of thermal conductivity, thermal diffusivity, and specific heat capacity in accordance with ISO 22007-2:2022 [37]. Dielectric breakdown strength was measured following the ASTM D149 standard [38] using a variable-frequency AC dielectric strength tester (HYYD-50 kV). A sphere-plate electrode configuration (stainless steel, sphere diameter of 6 mm, and plate diameter of 25 mm) was employed. All measurements were performed in transformer oil at room temperature to prevent surface flashover. A sinusoidal AC voltage at 50 Hz was applied with a constant ramp rate of 0.5 kV/s. Sample thickness was precisely controlled at 100 ± 5 μm using a micrometer caliper. For each composite composition, at least ten specimens were tested to ensure statistical reliability. Characteristic breakdown strength and shape parameter (β) were determined using a two-parameter Weibull distribution analysis (IEEE Std 930-2004) [39]. The cumulative failure probability *P*(*E*) is given by *P*(*E*) = 1 − exp[−(*E*/*E*_0_)*^β^*], where *E* is the measured breakdown field and *E*_0_ is the scale parameter. Data were linearized by the maximum likelihood estimation method, and the confidence intervals were set at 95%. This protocol has been widely adopted for evaluating electrical insulation of polymer nanocomposites, as recently discussed for polyurethane-based systems [40].

## 3. Results and Discussion

**Figure 2:**
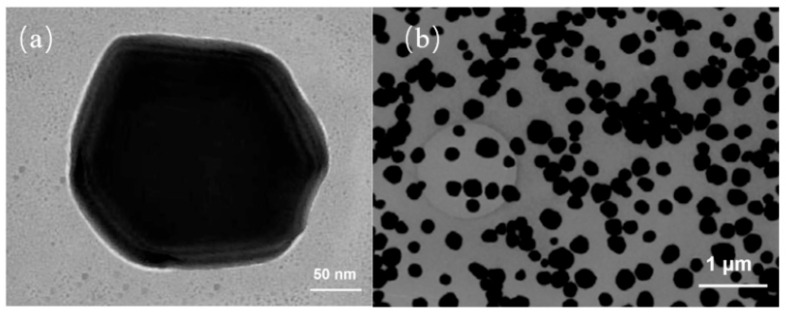
SEM images of AgNPs at different magnifications: (**a**) single particle and (**b**) particle aggregate.

The microstructure and morphology of the AgNP-BNNS/EP composites were characterized using SEM, as presented in Figure 2. Figure 2a reveals that the AgNPs exhibit a quasi-hexagonal shape with an average diameter of approximately 230 nm, markedly larger than the typical BNNS dimensions reported in prior studies [41]. Filler morphology plays a critical role in governing dispersion within the polymer matrix, and different geometric shapes (e.g., granular, flaky, or fibrous) display distinct dispersion behaviors. In the present system, the combination of granular AgNPs and flaky BNNSs yields a more homogeneous filler dispersion than that observed in single-filler systems (Figure 2b). The hybrid filler arrangement modifies the thermal conduction network and alters the spatial distribution of each filler type in the epoxy. Furthermore, the rigid particles act as stress-concentration sites, which increases the inter-particle distance and suppresses filler agglomeration. In summary, the distinct morphologies of AgNPs (granular) and BNNSs (flaky) synergistically improve their mutual dispersion in the epoxy matrix, and the incorporation of AgNPs effectively promotes the dispersion of BNNSs within the composite.

**Figure 3:**
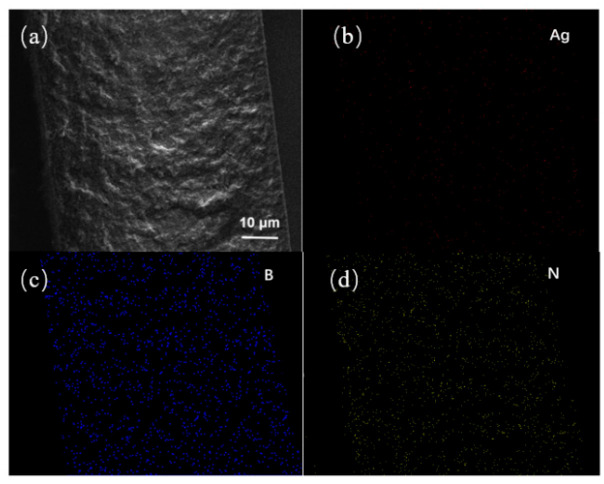
SEM images of (**a**) cross-sectional morphology of AgNP-BNNS/EP and (**b**–**d**) the element plane map of Ag, B and N.

Next, the cross-sectional morphology of the composite containing 20 wt% BNNSs and 1 wt% AgNPs was examined by SEM, as shown in Figure 3a. The composite displays a flat surface with a thickness of approximately 100 μm, which ensures the accuracy of subsequent measurements. EDS elemental mapping was performed on the same cross-section to visualize the dispersion of Ag, B, and N, with the corresponding distribution maps presented in Figure 3b–d. The Ag signal shows lower intensity and a more uniform distribution than the B and N signals, consistent with its lower loading and effective dispersion. In contrast, the B and N signals display similar distribution patterns and intensities, confirming the uniform dispersion of BNNSs throughout the matrix. Overall, the Ag content is notably lower than that of B and N, and both AgNPs and BNNSs are well dispersed in the epoxy. These results confirm the successful preparation of the hybrid composite material.

**Figure 4:**
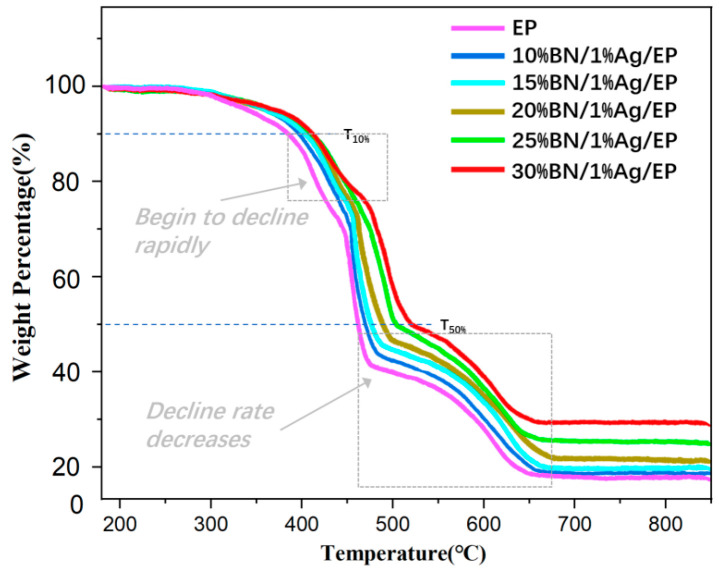
TGA curves of neat EP and AgNP-BNNS/EP composites with 1 wt% AgNPs and varying BNNS content.

Figure 4 presents the thermogravimetric (TGA) curves of neat EP and AgNP-BNNS/EP composites with a fixed 1 wt% AgNP content and varying BNNS loadings. As observed, neat EP exhibits the fastest mass loss and lowest residual mass across the entire temperature range, whereas all composites display slower degradation and higher char yields. Moreover, the onset of rapid decomposition is progressively delayed, and the char residue at 800 °C increases systematically with higher BNNS loadings. These results indicate that the incorporation of BNNSs progressively enhances the thermal stability of the composites. All samples undergo rapid weight loss starting around 400 °C, corresponding to the thermal degradation of the epoxy matrix, where chain scission and crosslink rearrangement occur. For instance, the composite with 30 wt% BNNSs retains 94% of its mass at 400 °C, compared to 88% for neat EP. Notably, the weight-loss rate slows considerably around 500 °C, likely because most of the epoxy has decomposed by this stage, while the thermally stable BNNS remains intact, moderating further mass loss. Additionally, uneven filler dispersion or local aggregation may induce localized thermal stress at high temperatures, further influencing the weight-loss behavior. The improved thermal stability of the AgNP-BNNS/EP composites can be attributed to three synergistic mechanisms [42,43,44]. First, a BNNS acts as an effective physical barrier that slows the diffusion of volatile degradation products and suppresses heat transfer. Second, AgNPs serve as thermal bridges between neighboring BNNSs, reducing interfacial thermal resistance and facilitating the formation of continuous 3D thermally conductive networks, which promote uniform heat dissipation and delay the onset of thermal degradation. Third, the hybrid fillers restrict the segmental mobility of the epoxy molecular chains, raising the glass transition temperature and improving the overall thermal stability. Additionally, the BNNS-induced increase in thermal degradation activation energy suggests a more stable composite system [6]. Taken together, a BNNS functions as an effective thermal barrier and stabilizer, and its synergistic interaction with AgNPs in forming a thermally conductive network is key to suppressing polymer degradation and enhancing char formation.

Table 1 summarizes the characteristic thermal decomposition temperatures (T_5%_, T_10%_, and T_50%_) of neat EP and AgNP-BNNS/EP composites with a fixed AgNP content of 1 wt% and varying BNNS loadings. As the BNNS content increases from 0 to 30 wt%, all three temperatures consistently rise, demonstrating enhanced thermal stability. For example, T_50%_ increases from 462.15 °C for neat EP to 533.28 °C for the composite with 30 wt% BNNSs, a gain of approximately 71 °C. Correspondingly, T_5%_ improves from 332.20 °C to 402.49 °C, and T_10%_ from 385.32 °C to 439.41 °C. These systematic shifts indicate that higher BNNS loading not only delays the onset of degradation and raises the temperature for substantial mass loss but also enhances the overall thermal conductivity of the composite. This promotes more efficient heat dissipation and reduces localized heat accumulation within the matrix, thereby contributing to the observed thermal stabilization.

**Figure 5:**
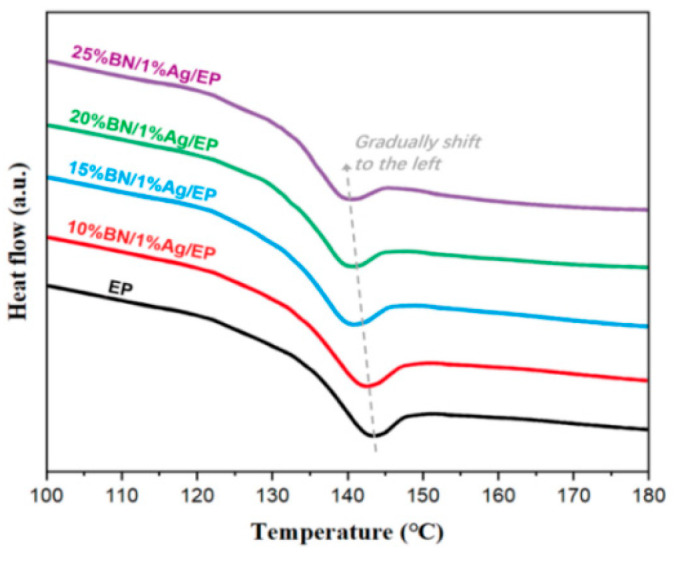
DSC curves of neat EP and AgNP-BNNS/EP composites with 1 wt% AgNPs and varying BNNS content.

Figure 5 presents the differential scanning calorimetry (DSC) curves of neat EP and AgNP-BNNS/EP composites with 1 wt% AgNPs and varying BNNS loadings (10–25 wt%). All samples exhibit a distinct step-like baseline shift characteristic of the glass transition of the crosslinked epoxy network, with no melting endotherms, confirming the amorphous thermosetting nature of the matrix. As BNNS content increases, the glass transition temperature (*T*_g_) progressively decreases from approximately 145 °C for neat EP to about 125 °C for the 25 wt% BNNS composite, accompanied by an increase in the step height. This behavior is attributed to a reduction in crosslinking density induced by the fillers, where BNNSs and AgNPs physically hinder contact between epoxy oligomers and the curing agent during network formation, thereby decreasing the number of effective crosslinking sites [45]. Concurrently, the endothermic peak associated with the relaxation of the crosslinked network becomes broader and less distinct. These changes indicate that the fillers disrupt the uniformity of the epoxy network [46]. Lower crosslink density increases free volume and chain mobility, thereby lowering the glass transition temperatures, as confirmed by the complementary dynamic mechanical analysis and equilibrium swelling experiments [47]. Thus, the DSC data consistently demonstrate that the introduction of hybrid fillers systematically lowers *T*_g_ by reducing the crosslinking density of the epoxy matrix.

**Figure 6:**
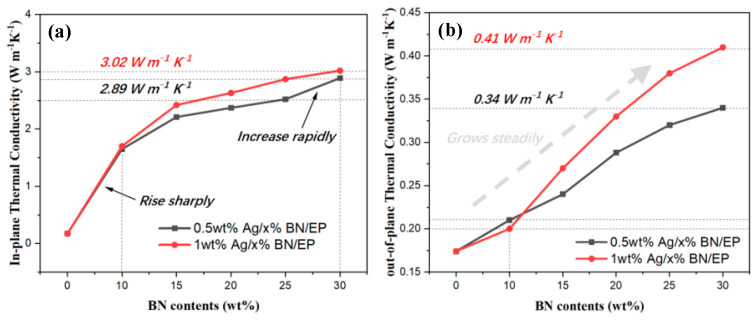
Thermal conductivities of the composites with 0.5 wt% and 1 wt% AgNP loadings: (**a**) in-plane and (**b**) out-of-plane.

To further evaluate the effect of filler content on thermal conductivity, a key performance metric in this study, the thermal conductivity of the composites was measured using an LFA447 Nanoflash^®^ system, Germany, as shown in Figure 6. Without BNNS addition, the in-plane and out-of-plane thermal conductivities of AgNP-only composites remain low (approximately 0.174 W·m^−1^·K^−1^ and 0.17 W·m^−1^·K^−1^, respectively) regardless of the AgNP loading, indicating that isolated AgNPs alone cannot form effective heat transport pathways in the epoxy matrix. When BNNS content reaches 10 wt%, a significant increase in in-plane thermal conductivity is observed (Figure 6a). At this loading, the composite with 1 wt% AgNPs exhibits a slightly higher in-plane thermal conductivity (1.7 W·m^−1^·K^−1^) than that with 0.5 wt% AgNPs (1.65 W·m^−1^·K^−1^). For the 0.5 wt% AgNP series, in-plane thermal conductivity increases gradually until BNNSs reach 25 wt%, then rises rapidly to 2.89 W·m^−1^·K^−1^ at 30 wt% BNNSs, which is higher than values reported in many recent studies on BN-based epoxy composites [42]. This accelerated enhancement likely results from the formation of abundant AgNP-BNNS thermal bridges above a percolation threshold, establishing a robust conductive network that reduces interfacial resistance and enhances phonon transport [43]. As phonons travel along the high-conductivity basal planes of BNNSs, their transport across adjacent BNNS interfaces is facilitated by AgNPs acting as thermal bridges, thereby converting isolated BNNS islands into a continuous network. In contrast, the composite with 1 wt% AgNPs exhibits a more gradual increase in in-plane thermal conductivity across the entire BNNS range, benefiting from well-dispersed AgNPs that aid heat transfer through more uniformly distributed bridging contacts [48]. However, the growth rate of in-plane thermal conductivity gradually declines at higher BNNS loadings. This slowdown can be attributed to increased filler agglomeration at elevated filler contents, which diminishes the advantage of the high aspect ratio of BNNSs, disrupts the continuity of the thermal conduction network, and impedes efficient heat flow [49]. Additionally, the percolation-like behavior observed here aligns with findings that hybrid filler systems can surpass the performance of single-filler composites by establishing continuous heat transfer paths, and facilitate phonon transport across the composite [36]. Collectively, the enhanced in-plane thermal conductivity originates from the synergistic effect of the hot-pressing process during curing and the intrinsically high thermal conductivity of the fillers [50,51].

**Figure 7:**
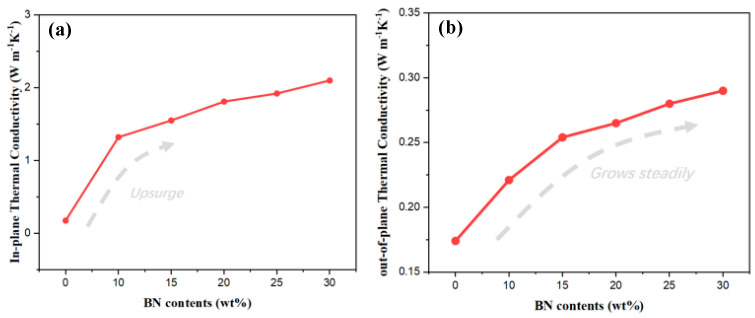
Thermal conductivities of the pure EP and BNNS/EP composites: (**a**) in-plane and (**b**) out-of-plane.

Figure 6b presents the out-of-plane thermal conductivity of the composites. For the 0.5 wt% AgNPs, the maximum out-of-plane thermal conductivity reaches only 0.34 W·m^−1^·K^−1^, lower than that achieved with 1 wt% AgNPs. For composites containing 1 wt% AgNPs, however, the out-of-plane thermal conductivity rises steadily from 0.17 to 0.41 W·m^−1^·K^−1^ with increasing BNNS loading, consistent with previous reports [36]. This discrepancy is primarily attributed to the synergistic interplay between the two fillers in the out-of-plane direction, combined with the flat geometry of the prepared samples (~100 µm) [43]. When AgNPs are uniformly dispersed, they are more likely to establish contact with BNNSs along the vertical (out-of-plane) direction than the lateral direction (in-plane), because the high-aspect-ratio BNNSs tend to align in-plane during hot pressing, leaving their edges and surfaces available for AgNP bridging in the thickness direction [52]. Consequently, the AgNP concentration largely governs the number of AgNP-BNNS thermal bridges formed within the material, which directly influences vertical phonon transport and the continuity of heat-flow pathways, thereby controlling the out-of-plane thermal conductivity. It is worth noting that the slight decrease in out-of-plane thermal conductivity observed for the 1 wt% AgNP composite at 10 wt% BNNSs may be ascribed to stochastic fluctuations in the formation of thermally conductive networks at low filler contents, where the filler concentration remains insufficient in establishing a continuous percolation pathway through the thickness [48]. To further compare the thermally conductivity performance, the thermal conductivities of pure EP and BNNS/EP composites were also measured, as shown in Figure 7. Pure EP exhibits virtually the same in-plane and out-of-plane thermal conductivities as the AgNP-only composites. With increasing BNNS content, the in-plane and out-of-plane thermal conductivities of the BNNS/EP composites rise rapidly and then tend to saturation. Even at 30 wt% BNNSs, the maximum in-plane and out-of-plane thermal conductivities of the BNNS/EP composite reach only 2.1 and 0.29 W·m^−1^·K^−1^, respectively, markedly lower than those of the AgNP-BNNS/EP composites. These results further demonstrate that the enhanced thermal conductivity in the hybrid composites originates from the synergistic effect between AgNPs and BNNSs.

**Figure 8:**
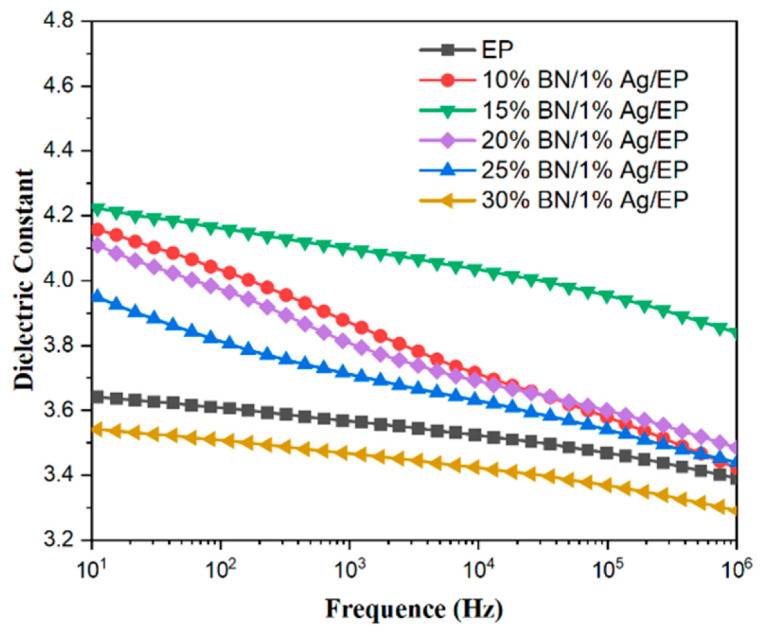
Dielectric constants of neat EP and AgNP-BNNS/EP with 1 wt% AgNPs and varying BNNS content.

To further evaluate the effect of BNNS filler on the dielectric constant of the composites, the frequency-dependent dielectric constant (ε′), AC conductivity (*σ*), and dielectric loss (tan *δ*) were measured at room temperature. As shown in Figure 8, the dielectric constant reaches a maximum of 4.17 at 100 Hz for the composite with 15 wt% BNNSs and 1 wt% AgNPs. At the same frequency, neat EP exhibits a dielectric constant of only 3.6, while all composites with other BNNS loadings show values below 4.17, indicating that an appropriate BNNS loading enhances the dielectric performance. This enhancement is primarily attributed to three synergistic mechanisms: Maxwell–Wagner–Sillars (MWS) interfacial polarization, microcapacitor effects, and charge accumulation at the AgNP/EP and BNNS/EP interfaces [53,54,55,56,57]. First, the significant conductivity mismatch between conductive AgNPs and the insulating EP matrix causes charge carriers to migrate and accumulate at the filler–polymer interfaces under an alternating electric field, generating strong MWS interfacial polarization. Second, adjacent AgNPs separated by thin EP layers form numerous microcapacitors, where each AgNP acts as an electrode and the intervening EP as the dielectric, efficiently storing charge and increasing ε′, and the presence of BNNSs prevents direct AgNP contact, thereby preserving insulation and limiting loss. Third, significant charge accumulation at both AgNP/EP and BNNS/EP interfaces, arising from atomic-scale charge redistribution, further reinforces overall polarization. Thus, incorporating conductive fillers into the epoxy matrix effectively increases the dielectric constant of the composite.

**Figure 9:**
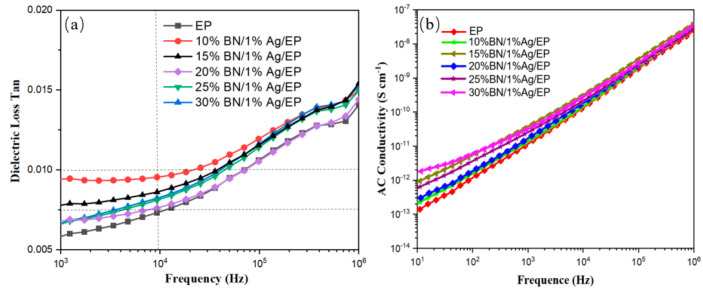
Dielectric loss and AC conductivity of neat EP and AgNP-BNNS/EP with 1 wt% AgNPs and varying BNNS content: (**a**) tan *δ* and (**b**) σ.

Figure 9 presents the tan *δ* and *σ* of neat EP and AgNP-BNNS/EP composites. As shown in Figure 9a, tan *δ* of all samples increases slightly with frequency, a typical frequency-dependent behavior arising from polarization lag behind the alternating electric field at higher frequencies. All measured tan *δ* values remain below 0.03 across the entire frequency range, confirming that the composites retain excellent electrical insulation with no significant conductive loss introduced by the fillers. At high frequencies, the loss curves converge, as charge motion becomes confined to short-range hopping governed by the intrinsic relaxation of the epoxy matrix. Additionally, dielectric loss also shows a non-monotonic dependence on BNNS loading. At 10 wt% BNNSs, tan *δ* slightly exceeds that of neat EP, which is attributed to enhanced interfacial polarization promoted by conductive AgNPs. With a further increase in BNNS content, the loss returns to a level comparable to neat EP, because the insulating BNNSs act as barriers to long-range charge migration and provide charge-trapping sites, thereby suppressing conduction-related loss. Overall, tan *δ* in these composites is determined by competition between the loss-enhancing interfacial polarization from AgNPs and the loss-suppressing barrier/trapping effect from BNNSs.

Figure 9b depicts the *σ* of neat EP and the composites as a function of frequency from 10^1^ to 10^6^ Hz. For all samples, *σ* increases with frequency, consistent with previous reports [58]. Across the entire BNNS loading range (10–30 wt%), the *σ* values of the composites are slightly higher than those of neat EP, but their absolute conductivities remain very low. For example, at 10^3^ Hz, neat EP exhibits σ ≈ 1.5 × 10^−11^ S·cm^−1^, while all composites maintain *σ* below 1.0 × 10^−10^ S·cm^−1^. Although this difference corresponds to approximately one order of magnitude, both values fall well within the insulating regime for practical applications. At low frequencies (10^1^–10^2^ Hz), the conductivity fluctuates without a clear trend with BNNS loading, likely due to local filler agglomeration and associated defects at higher BNNS contents. At high frequencies (>10^4^ Hz), the *σ* values of all samples converge, indicating that the conduction mechanism becomes dominated by the epoxy matrix regardless of filler loading. Notably, no sharp conductivity increase is observed even at 30 wt% BNNSs, demonstrating that the conductive AgNPs remain isolated and do not form percolating pathways. This is attributed to the insulating BNNS layers, which act as physical barriers that prevent direct AgNP contact and disrupt potential conductive networks [59]. Consequently, charge transport is governed by the epoxy matrix, where conductivity rises steadily with frequency due to enhanced hopping and polarization contributions. These results collectively confirm that the hybrid filler system preserves the excellent electrical insulation of the base polymer, with only a modest increase in *σ* that remains within acceptable limits for insulating composites.

**Figure 10:**
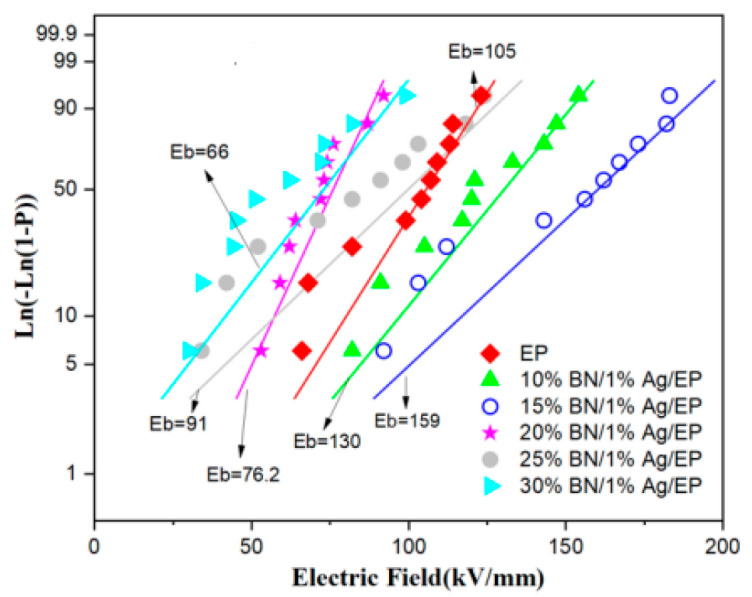
Breakdown strength of neat EP and AgNP-BNNS/EP with 1 wt% AgNPs and varying BNNS content.

To further evaluate the insulating properties of the composites, the breakdown strength was analyzed using Weibull statistics, as shown in Figure 10. The AgNP-BNNS/EP composites exhibit a clear dependence of breakdown strength on filler loading, following a non-monotonic trend. The breakdown strength initially increases with BNNS content, reaching a maximum of 159 kV/mm at 15 wt% BNNSs, significantly higher than that of neat EP (105 kV/mm). Correspondingly, the Weibull shape parameter (β), which reflects the reliability and uniformity of breakdown, is expected to be lowest at this optimal composition. Beyond this point, further BNNS addition reduces the breakdown strength, which declines to 66 kV/mm at 30 wt% BNNSs. This behavior can be explained by the microstructure evolution within the composites. At lower BNNS contents (≤15 wt%), the hot-pressing process promotes in-plane alignment of the high-aspect-ratio BNNSs. This aligned structure effectively blocks conductive pathways formed by AgNPs, suppresses the initiation and propagation of electrical trees, and thereby enhances breakdown strength. However, when the BNNS content exceeds 20 wt%, local filler agglomeration occurs, resulting in a less uniform and less effective insulating barrier, which ultimately compromises the breakdown performance.

## 4. Conclusions

In summary, we have successfully demonstrated the fabrication of epoxy-based composites incorporating a hybrid filler system of AgNPs and BNNSs. Systematic characterization reveals that the synergistic interplay between the two fillers effectively enhances both thermal transport and dielectric performance while preserving the inherent electrical insulation of the epoxy matrix. Specifically, the introduction of a minimal amount (1 wt%) of AgNPs significantly promotes the uniform dispersion of BNNSs within the polymer matrix. This improved distribution reduces interfacial polarization losses and raises the dielectric constant to 4.17 at 100 Hz for the composite with 15 wt% BNNSs, markedly higher than that of neat epoxy. Moreover, at a higher BNNS loading of 30 wt% combined with the 1 wt% AgNPs, the composite exhibits outstanding thermal conductivity: 3.02 W·m^−1^·K^−1^ in-plane and 0.41 W·m^−1^·K^−1^ out-of-plane. This anisotropic thermal enhancement is accompanied by superior electrical insulation, with low-frequency electrical conductivity remaining on the order of 10^−9^ S·cm^−1^ at 1000 Hz, even with the inclusion of metallic nanoparticles. Collectively, these findings underscore the efficacy of the 0D/2D hybrid filler design in achieving a well-balanced trade-off among thermal conductivity, dielectric response, and electrical insulation. The proposed strategy offers a practical and scalable pathway for developing next-generation high-performance polymer composites tailored to advanced electronic packaging requirements.

## Figures and Tables

**Table 1:** Characteristic thermal decomposition temperatures of neat epoxy and AgNP-BNNS/EP composites with varying BNNS contents.

Samples	T_5%_ (°C)	T_10%_ (°C)	T_50%_ (°C)
EP	332.20	385.32	462.15
10%BN/1%Ag/EP	357.61	407.10	483.64
15%BN/1%Ag/EP	360.90	415.52	496.96
20%BN/1%Ag/EP	379.21	428.88	508.20
25%BN/1%Ag/EP	387.28	437.11	527.21
30%BN/1%Ag/EP	402.49	439.41	533.28

## Data Availability

The original contributions presented in this study are included in the article. Further inquiries can be directed to the corresponding authors.
